# The Effect of Sport Practice on Enhanced Cognitive Processing of Bodily Indices: A Study on Volleyball Players and Their Ability to Predict Hand Gestures

**DOI:** 10.3390/ijerph18105384

**Published:** 2021-05-18

**Authors:** Giovanni Ottoboni, Roberto Nicoletti, Alessia Tessari

**Affiliations:** 1 Department of Psychology, University of Bologna, 40127 Bologna, Italy; giovanni.ottoboni@unibo.it; 2 Department of Philosophy and Communication Studies, University of Bologna, 40122 Bologna, Italy; roberto.nicoletti@unibo.it

**Keywords:** action prediction, motor expertise, action directionality, body representation, volleyball, sport

## Abstract

To program proper reactions, athletes must anticipate opponents’ actions on the basis of previous visuomotor experience. In particular, such abilities seem to rely on processing others’ intentions to act. We adopted a new approach based on an attentional spatial compatibility paradigm to investigate how elite volleyball players elaborate both spatial and motor information at upper-limb posture presentation. Forty-two participants (18 volleyball players and 17 nonathlete controls assigned to Experiments 1 a and b, and eight basketball players assigned to Experiment 2) were tested to study their ability to process the intentions to act conveyed by hands and extract motor primitives (i.e., significant components of body movements). Analysis looked for a spatial compatibility effect between direction of the spike action (correspondence factor) and response side for both palm and back of the hand (view factor). We demonstrated that volleyball players encoded spatial sport-related indices from bodily information and showed preparatory motor activation according to the direction of the implied spike actions for the palm view (Experiment 1; hand simulating a cross-court spike, *p* = 0.013, and a down-the-line spike, *p* = 0.026) but both nonathlete controls (Experiment 1; both *p* < 0.05) and other sports athletes (basketball players, Experiment 2; *p* = 0.34, only cross-court spike) did not. Results confirm that elite players’ supremacy lies in the predictive abilities of coding elementary motor primitives for their sport discipline.

## 1. Introduction

The ability to anticipate events and actions in sports is essential to interact with the environment effectively [1,2,3]. In addition to being a necessary ability in daily life, its role is paramount in sport situations where good athletes intercept the trajectory of moving objects by anticipating either opponents’ or playmates’ actions. Due to the short time characterising the sport actions, excellence is not only based on good motor technical abilities but also on the ability to understand other players’ moves from partial, or even missing, visual information through top-down modulation [4,5]. Some behavioural studies revealed differences between athletes and nonathletes in terms of processing abilities for both visuo-perceptual and motor skills. In ball games, for example, experienced athletes can anticipate “where” and “when” the ball will be thrown on the basis of information extracted from movements of the opponent, even before the ball has begun its trajectory [6,7,8]. This is a necessary ability as athletes, using their perceptual expertise, need to intercept the ball at the right place and at the right time as there are frequent exchanges in ball possession and players must respond to them by making decisions whether they are in possession or not of the ball [9].

Advantages of athletes over nonathletes also emerge in other cognitive domains. For example, expert athletes recognise and store complex patterns of actions better than either novices or nonathletes, as well as anticipate perceptual strategies [10]. It is long known that this ability is based on a more expansive visuomotor repertoire used for both visual perception and motor execution and built over years of practice [11,12,13]. Other studies also showed different strategies in focusing on or ignoring relevant vs. irrelevant information between expert players and novices [3,14,15,16]. Regarding open-skill sports, the perceptual–cognitive expertise in elite volleyball players led to superior performance speed in executive control and visuospatial attentional processing tasks [17], and a positive correlation between cognitive and motor functioning was found [18]. In young volleyball athletes, Trecroci and colleagues [18] reported a positive correlation between cognitive functions (such as executive control and perceptual speed) and sport-specific physical performances.

A cognitive advantage also emerged in the visual attention domain for team sports. These disciplines require players to control either the activities/actions or the positions of many players simultaneously and understand how these positions will change over time. For example, athletes have greater sensory–cognitive skills related to their specific sport domain [1] and are able to predict the forthcoming direction and force of an opponent’s stroke better then novices [11]. Volleyball athletes also showed superior performance in executive control tasks and a visuo-spatial attentional processing task compared to controls, suggesting a sport–cognition relationship [17]. This is not surprising as athletes often use and process early visual cues, such as ball flight (in fast ball sports), to predict the forthcoming event and identify the opponent’s action. However, some authors based their results on the ‘cognitive component skills theory’, suggesting superior innate cognitive skills in elite athletes compared to subelite expert players, already at a very young age [19,20,21,22,23,24].

Many researchers suggested that the ventral and the dorsal brain systems [25,26,27,28] may play an essential role in visual anticipation in fast ball sports [6,29,30,31,32,33,34]. The ventral system holds explicit knowledge about what the environment offers for action and specifies the location, motion, and size of an object in relation to other objects. This information enables the athletes to identify the appropriate action the situation affords (e.g., it may gather information on whether a down-the-line or cross-court shot is the most appropriate action). On the contrary, the dorsal system is involved in action planning and detects information about the location, motion, orientation, and size of an object relative to the observer/agent. Van der Kamp and colleagues [35] proposed that the ventral system plays a crucial role before movement onset, whereas the dorsal system dominates after movement onset. Indeed, while observing an opponent’s action, the ventral system gathers information about what (counter-)action the situation affords (e.g., in volleyball, the athletes may decide a return stroke or to hit the ball) on the basis of the opponent’s kinematics [4,34,36], their location on the court, and the ball flight. The dorsal system allows the athletes to prepare and execute an adequate response action by controlling the movement execution. If we consider fast ball sports, like volleyball, it is evident that athletes begin to move before ball release and can initially plan a movement on the basis of the opponent’s kinematics information.

Researchers have suggested that the action–observation network plays an essential role during such anticipation in expert athletes (at least volleyball and tennis); in particular, the superior parietal lobe and supplementary motor area positively correlate with anticipation abilities, suggesting that better perceptual–motor representations have improved over years of training [37]. Tomasino and colleagues [38] also demonstrated that expert volleyball players, compared to novices, use motor simulation (involving activation in the left primary motor cortex hand area and premotor cortex) while judging sentences, describing possible technical volleyball-specific motor acts but not impossible ones, suggesting a role of domain-specific expertise and interaction between motor and visual imagery at a more general conceptual stage.

### This Study

In this study, we focus on athletes’ expertise to anticipate and make predictions from partial or incomplete sources of visual information. In particular, we investigate the ability to predict the outcome of sports actions and concentrate on two crucial aspects that have been left aside so far: (1) what features are *implicitly* and *unconsciously* elaborated while observing actions (thus, without deliberately trying to predict or respond to the result of an action); (2) what is the motor and spatial information conveyed by single bodyparts and postures during such an unconscious and implicit elaboration. Moreover, we use a new attentive paradigm allowing us to study the implicit (first aspect) elaboration of bodypart postures (second aspect).

So far, action prediction and anticipation have usually been studied via the “temporal occlusion paradigm”, where participants *explicitly* and *consciously* anticipate the results of visually displayed actions [30]. Whole-body actions are shown for a partial amount of the entire activity; thus, the conveyed information increases dynamically with time. The action is then masked, and participants must predict action results [10,39,40,41].

On the contrary, in this study, we used an attentive, *implicit* task (the sidedness paradigm [42]), and we did not present action videos but only static pictures of body parts. The method offers the possibility to understand whether athletes retain specific coding skills allowing them to extract “motor primitives” from body parts salient for their sport. Motor primitives are the fundamental kinematic and kinetic building components of body movements, developed ontogenetically, and an observer can subsume the results of a movement from them [43]. In the case of volleyball players, as a function of their sport-specific expertise, they might have developed the ability to extract the results of an action from the cue conveyed by hand/forearm postures. During the match, athletes direct their attention to the opponent’s upper-limb stance to anticipate the ball’s next direction. Such an ability should persist even during the presentation of static images. Indeed, it has been shown that showing static images of whole-body or single-body parts, conveying the impression that they are about to move (i.e., *implied motion*), activates medial–temporal brain areas governing the elaboration of real movements and the fronto-parietal network underpinning both action planning and comprehension [44,45]. These results suggest that the cognitive system creates complete dynamic motion representations from those developed through visual and motor experiences. Frey [46] proposed that previously seen and/or performed actions are stored in motor long-term memory [47,48], recalled at the view of action-related visual cues, and then matched to predict action results. Thus, the cognitive system is able to recall complete and dynamic motion representations starting from visual motion-related cues. To investigate the ability to read kinematic information from simple motor posture (motor primitives), neither the ball nor the athletic environment was shown. In this way, we could extrapolate athletes’ ability to “read” and “predict” action goals from purely visuo-kinematic bodily aspects independently of the context.

## 2. Experiment 1

We tested if volleyball athletes could process the information of an “implied” spike action (instead of real video action) and predict the resulting direction applied to the ball by presenting hand postures. If the ability to “read” actions is based on recognising motor primitives rather than decoding an entire action, we might be able to measure the minimum amount of information necessary to encode a response action. To achieve our goal, we used a modified version of the Simon task [49], already used to study implicit hand processing and orientation of attention [42,50,51,52]. This task allows for investigating how attention is oriented in space and whether such an attentional shift preactivates spatially oriented motor responses (see also [3] in boxing athletes). Indeed, the *attention shift hypothesis* [53,54] conceived that the attentional selection of a stimulus location in space primes its corresponding motor response, i.e., actions are facilitated towards attended spatial locations because attentional orienting towards a location overlaps with the preparation of actions towards it (i.e., a spatial compatibility effect emerges between spatial location of attention and response spatial position).

We predicted that volleyball players should process the implied motion conveyed by hand postures and the following information on the direction of the potential spike action. They should code the final position where the ball arrives, move automatically, implicitly direct their attention toward that spatial side, and activate/prepare for the adequate response action (i.e., the reception of the ball) according to the *attention shift hypothesis* [53,54]. They should be faster in performing the task when responding with the hand on the same side of the implied spike action. However, we also expected a difference in the coding of the two hand views: the palm and the back. The palm view, being more salient in the volleyball context—as it represents an opponent’s hand—should be more salient than back-viewed hands, being interpreted as one’s own hands or as a teammate’s hand. The backs of hands were, thus, not expected to induce any action direction processing. Moreover, we did not expect such an effect in nonathletes as they have not developed such an ability to extract action directionality cues from hand postures.

### 2.1. Methods

#### 2.1.1. Participants

A convenience sample of 18 right-handed male volleyball players belonging to an elite team (A1 series) was tested. Nine athletes (25.7 years old, SD = 4.85) were randomly assigned (simple randomisation) to Experiment 1a and nine (25 years old, SD = 5.36) were randomly assigned to Experiment 1b.

These players had been practising volleyball from 5 to 20 years (mean 12.3, SD = 4.5). The study was conducted during the second half of the competitive season, from April to May. They trained twice per day from 4 to 6 days per week, with each training session lasting 3 h on average.

Moreover, eight right-handed male nonathletes were assigned to Experiment 1a (25.5 years old, SD = 1.83) and nine male non-athletes were assigned to Experiment 1b (23.56 years old, SD = 3.73). All participants reported having normal or correct-to-normal vision and were right-handed.

A power analysis was conducted on the interaction view × correspondence according to Ottoboni et al. [42] (Experiments 2A, 2B, and 3) and Tessari et al. [52] (Experiment 1). Such an interaction represents the relevant interaction to check whether an automatic and implicit shift of attention has been performed towards that spatial side of the action and whether a spatially facilitated manual motor response has been preactivated (spatial compatibility effect according to the attention shift hypothesis). We used G *Power (HHU, Düsseldorf, Germany) [55]. With a mean effect size of Cohen’s *d* = 1.14 (calculated for the view × correspondence interaction in the papers mentioned above), alpha = 0.05, and power of 0.80, we needed seven participants.

#### 2.1.2. Ethics Statement

The study was carried out in accordance with the Helsinki Declaration of 1975 and was approved by the ethics committee of the University of Bologna (approval n. 142212).

#### 2.1.3. Apparatus and Procedures

**Figure 1:**
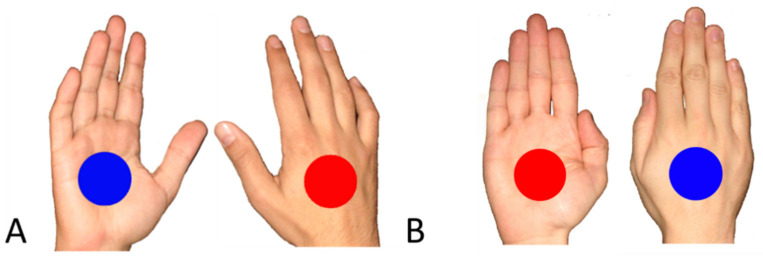
Stimuli used in Experiments 1 (**A**,**B**) and 2 (**A** only) are shown.

Stimuli were photographs (23° × 9°) of right and left hands in palm and back view, 30° rotated along their ulnar axis (Experiment 1a, see Figure 1A) or frontally presented (Experiment 1b, see Figure 1B). Oriented hands were used to simulate a cross-court spike, and frontal hands were used to simulate a down-the-line spike. The hand photographs were presented in the center of a computer screen (a ThinkPad laptop with a 21 inch screen), and a red or blue circle (4°) was superimposed in their center. Due to the implicit nature of the paradigm, participants were not required to pay attention or judge information about the hand posture but to judge and respond according to the colour of the circle by pressing one of two computer keyboard keys (“X” and “.”), respectively, on their left and right side, with the index fingers of their left and right hands. Participants were required to respond to the color of the circle as quickly and accurately as possible. The trial began with a central fixation cross for 1000 ms; then, the hand-circle stimulus lasted on the screen for 100 ms, and participants had up to 1000 ms to perform the color judgment response. Feedback about latency, errors, and omissions was presented for 1500 ms after response.

A total of 120 back-hand views and 120 palm-hand views were shown in two separated blocks for a total number of 240 trials. Both block order and colour-response side association were counterbalanced across participants. The presentation of the stimuli and the response collections were controlled using E-Prime 1.1 (Psychology Software Tool, Inc., Sharpsburg, USA) [56]. The software automatically recorded reaction times (RTs) and scored accuracy. Brief instructions were presented at the beginning of the experiments.

Hands were shown without their forearm to refrain the sidedness effect from emerging [42]. Indeed, evidence showed that hands or feet are referred to a body only if anatomo-physiological links, such as the forearm or ankle, are present [42,51,57] and follow the biomechanics laws of the human body [50,58].

#### 2.1.4. Statistical Analysis

Reaction times (RTs) 2.5 standard deviations above or below each participant’s mean for each condition were excluded from analyses for each condition following a standard filtering procedure for outliers [59].

Wrong responses were also excluded but not analysed due to the small data numerosity (5.83%). A 2 × 2 analysis of variance (ANOVA) for repeated measures with the within-subject factors view (back vs. palm of the hands) and correspondence (corresponding vs. noncorresponding pairings between action directionality and response side) was used to look for the significant interaction revealing the spatial compatibility effect based on directionality elaboration. We also used a between-group factor (athletes vs. nonathletes) to check whether a general RT difference exists between athletes and nonathletes, as already emerged in other studies (e.g., [17]). 

Corresponding pairings were those where the responding hand matched the direction of the spike action (e.g., an oriented palm-view right hand directing the ball to the right side, from the observer’s point of view, and a right-hand response—Experiment 1a). The noncorresponding pairings were those where the responding hand did not match the direction of the spike action implied by the hand stimulus (e.g., frontally oriented palm-view right-hand, directing the ball to the left side of the observer, and right-hand response—Experiment 1b).

Effect sizes (ESs) were computed for each significant analysis; ES was interpreted as null (<0.2), small (0.2–0.5), medium (0.5–0.8), large (0.8–1.20), or very large (>1.20).

The level of significance was set at *p* ≤ 0.05 for ANOVA. For comparisons, we used a one-tailed *t*-test with no correction, as the effect was predicted according to the literature (e.g., Freyd, 1983; Nicoletti and Umiltà, 1994; Stoffer, 1991; Tessari, Ottoboni, Mazzatenta, et al., 2012). Statistical analysis was performed using R 3.6.3 (R Foundation for Statistical Computing, Vienna, Austria).

#### 2.1.5. Results

We analysed the data for the two groups separately to check for a significant correspondence × view interaction based on a spatial compatibility effect between the direction of the action and the facilitated motor response.

**Figure 2:**
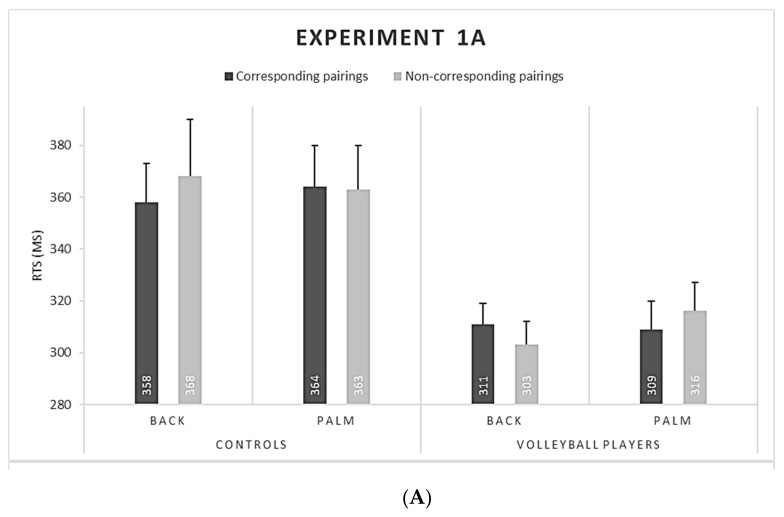

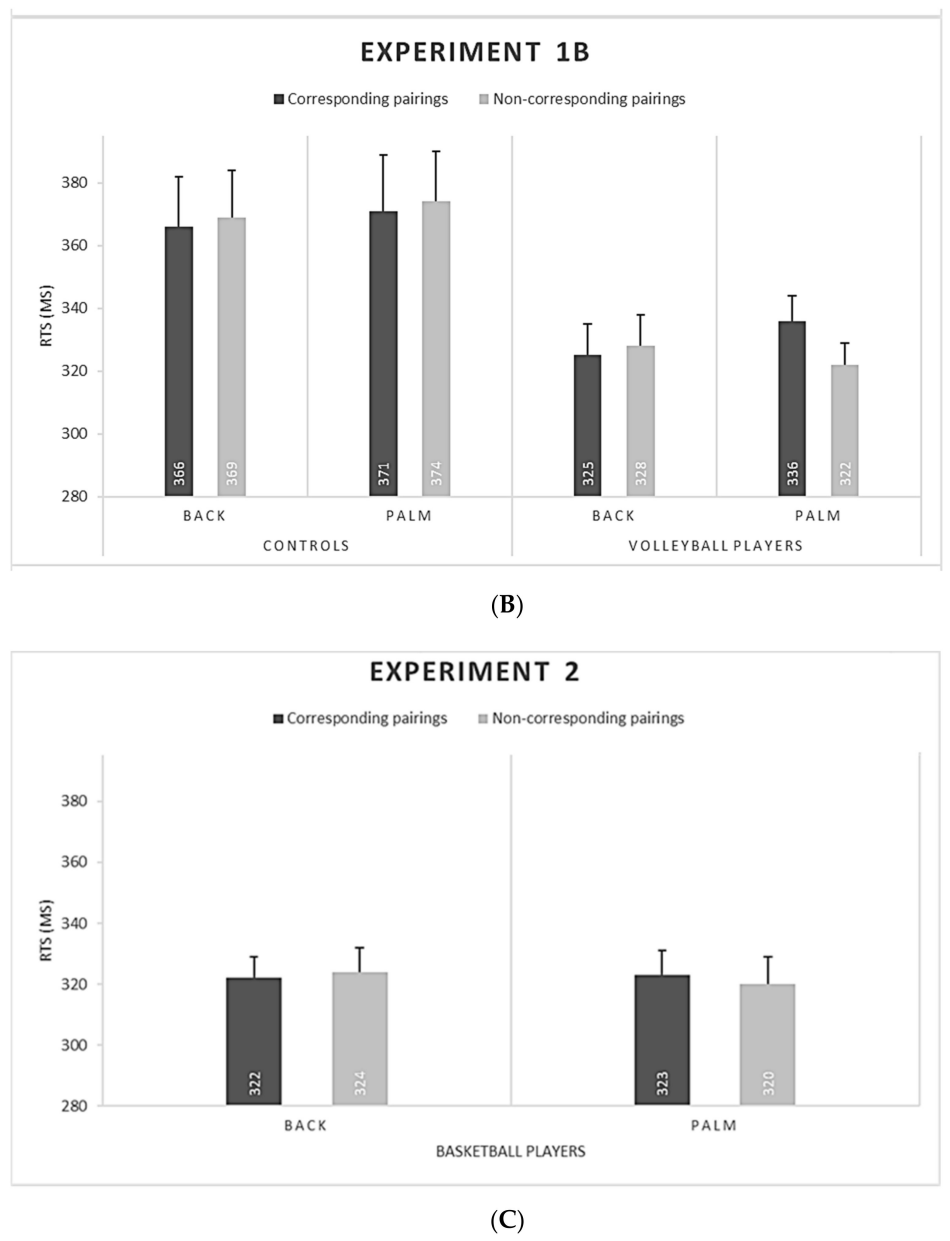
The graph shows RTs in Experiment 1a (**A**), Experiment 1b (**B**), and Experiment 2 (**C**), displayed according to view (back vs. palm) and correspondence (corresponding vs. noncorresponding pairings) for volleyball players, nonathletes, and basketball players.

In Experiment 1a, athletes did not show effects of view (F(1,8) = 0.73, η_G_^2^ = 0.008, *p* = 0.419) and correspondence (F(1,8) = 0.01, η_G_^2^ < 0.001, *p* = 0.99) factors, but their interaction was largely significant (F(1,8) = 10.14, η_G_^2^ = 0.020, *p* = 0.013, *d* = 1.12). Corresponding and noncorresponding pairings did not differ for back views (one-tailed *t*-test, *t*(8) = 1.70, *p* = 0.065). However, corresponding pairings were faster than noncorresponding ones for palm views (one-tailed *t*(8) = −1.92, *p* = 0.046). For nonathletes, both the two factors (view: F(1,7) = 0.01 η_G_^2^ < 0.001, *p* = 0.984; correspondence: F(1,7) = 0.96, η_G_^2^ < 0.001, *p* = 0.359) and their interaction were nonsignificant (F(1,7) = 1.30, η_G_^2^ < 0.01, *p* = 0.292) (see Figure 2A).

Overall, athletes (M = 310 ms, SE = 4.68) responded faster than nonathletes (M = 363 ms, SE = 8.46): Group, F(1,15) = 8.862, η_G_^2^ = 0.33, *p* = 0.010, *d* = 0.55.

In Experiment 1b, athletes showed faster RTs for corresponding (330 ms, SE = 6.34) than noncorresponding pairings (325 ms, SD = 6.03): F(1,8) = 9.22, η_G_^2^ = 0.010, *p* = 0.016, *d* = 1.02. No effect emerged for view (F(1,8) = 0.17, η_G_^2^ = 0.003, *p* = 0.688), but the interaction was significant (F(1,8) = 7.45, η_G_^2^ = 0.028, *p* = 0.026, *d* = 0.83). RTs did not differ between corresponding and noncorresponding pairings for back views (one-tailed *t*(8) = −1.14, *p* = 0.29), but corresponding pairings were faster than noncorresponding ones for palm views (one-tailed *t*(8) = 3.39, *p* = 0.004).

As shown in Figure 2B, nonathletes showed no significant effect or interaction, all *p* > 0.05 (view, F(1,7) = 1.15, *p* = 0.252, correspondence, F(1,7) = 0.60, *p* = 0.460, and view × correspondence, F(1,7) = 0.003, *p* = 0.966) (see also Experiment 1b in Ottoboni et al. [42] for similar results). 

An effect of group was found; athletes (328 ms, SE= 4.33) were faster than nonathletes (370 ms, SE = 7.74): F(1,17) = 5.71, η_G_^2^ = 0.25, *p* = 0.029, *d* = 0.32.

#### 2.1.6. Discussion

Only athletes showed a compatibility effect based on the direction of the attack action for palm hands (e.g., in Experiment 1a, an oriented right palm hand, directing a spike to the right, induced faster responses with the right hand; in Experiment 1b, a frontal right palm hand, presaging a down-the-line spike directed to the left, induced faster responses on the left). No effect emerged for back hands for both athletes and nonathletes (see also [42], Experiment 1b). Nonathletes showed no effect at all. Results suggest that highly experienced athletes, when presented with sport-salient stimuli (i.e., hands), can process the potential action direction that the hand might give to a ball. It is plausible that they recall the action of an opponent player and mentally simulate it by observing the hand’s postures. Due to their expertise, athletes become able to prepare for an adequate response action (e.g., a receipt) by implicitly reading body kinematics based on salient motor primitives for their sport. Practice might develop in athletes the predictive abilities about ball trajectories based on body postures even in the absence of an actual moving object. Indeed, in order to interact with a moving object, the cognitive system must create a perceptual model of the motion in the environment [60], and this is based on both visual experience and motor experience [61]. The significantly different patterns of results with palm and back views (athletes could get action directionality only for the palm view) suggest that visuo-perceptual experience plays an essential role in the action direction anticipation skill. The motor experience might also play a role in this elaboration, as the palm view activates the motor representation of the response to the attack action rather than a mirror representation of it (the latter would have also induced a directionality effect for the back views). However, in this automatic and implicit processing stage, visual expertise seems to play a more important role compared to the motor expertise, as the back view fails to induce an elaboration of the potential actions even though it can be easily referred to one’s own hands and generate an ownership effect, thereby inducing expert athletes to attribute it to themselves and to simulate spike actions and activate spatial codes on action directionality.

The salience of hand visual analysis in anticipating spike direction also finds support from Park’s study [62], demonstrating that expert volleyball players fixate longer on the spiker’s upper body, especially the arm area, as essential kinematical information to predict and anticipate the attack patterns and directions more effectively.

## 3. Experiment 2

In this experiment, we investigated if other athletes playing an open-skill sport in which hands are as important as in volleyball, i.e., basketball, can code action directionality conveyed by hand posture. In basketball, hands are continuously used, but movements/actions are not as “standardised” as in volleyball, and their posture continually changes according to the type of action to be performed (e.g., the shot, the dribble). Thus, basketball players use oriented hand actions to direct the ball; however, unlike volleyball, the opponent’s hand is not always clearly visible and is covered by the ball for a longer time than in volleyball.

Comparing volleyball to basketball players might allow us to understand whether results from Experiments 1 are due to the type of effectors usually used in the sport actions or other factors. Suppose that basketball athletes can also “read” others’ action intentions by extracting hand motor primitives to anticipate action outcomes accordingly to the opponent’s hand position. In that case, they should automatically direct their attention to the portion of the space where the implied action is directed to. If visual–motor experience plays the most important role, they might not show a directionality effect as found in volleyball players. However, if the motor experience of the very same actions is also important, they might recall the same mental processes involved in executing those actions, and they might recognise, understand dynamics, and prevent/anticipate the action results. Thus, motor expertise might allow them to anticipate the final position of the ball according to the shown action and orient attention accordingly, as shown by volleyball players in Experiment 1, by completing the missing visual information [63]. We predict that, due to the limited visual experience with the complete hand movement and less stereotypical actions, basketball players should not automatically code the implied action’s directionality.

### 3.1. Method

#### 3.1.1. Participants

Eighteen right-handed male basketball players belonging to an elite team were tested (23.78 years old, SD = 5.31) in Experiment 2 (years of activities, M = 12.4, SD = 5.5). They all had normal or correct-to-normal vision and were right-handed according to an Oldfield test (1981). They were used to training 8–10 times per week, with each session lasting 3 h on average.

#### 3.1.2. Apparatus and Procedures

Apparatus and procedure were identical to Experiment 1, and only oriented hands without the forearm were used (see Figure 1A). The same laptop and software were used for running the experiment and collecting the data. Data cleaning and statistical analysis followed the same procedure as in Experiment 1.

#### 3.1.3. Results

View and correspondence factors were analysed. The error rate was at 8.31%. As shown in Figure 2C, none of the factors or their interaction were significant: view (F(1,17) = 0.18, η_G_^2^ < 0.001, *p* = 0.674); correspondence (F(1,17) = 0.18, η_G_^2^ < 0.001, *p* = 0.674); view ×correspondence (F(1,17) = 1.11, η_G_^2^ < 0.001, *p* = 0.308).

We also compared their performance to that of volleyball players (specifically, Experiment 1a), adding a between-subject factor (basketball vs. volleyball). There was no difference between the two groups of athletes (F(1,25) = 0.94, η_G_^2^ = 0.032, *p* = 0.341).

#### 3.1.4. Discussion

No spatial compatibility effect emerged with basketball players, suggesting that these athletes could not automatically process the direction of the hand action to be performed. Two hypotheses can be put forward. First, basketball players may consider the visual information conveyed by the stimulus hands not informative in their sport. Second, hand positions are not as relevant as in volleyball because they are not informative for motor response planning, and they have not reached the stereotyped state of motor primitives. Indeed, in basketball, attention is not oriented toward the point in space where the hand action outcome is directed, and athletes better understand the game and move according to the situation and the surrounding environment’s conditions.

However, the absence of effect in the basketball players also favours a dominant role of visuo-perceptual experience in generating this phenomenon. The literature suggests that athletes use their previous visuomotor experience to simulate others’ actions, read body kinematics, and anticipate their future behaviour [35,64]. Thus, visual knowledge can be responsible for elaborating the predictive model of object motion and directionality; on the other hand, motor expertise can allow athletes to prepare the adequate behavioural response. It is important to highlight that, in this study, volleyball athletes formed anticipatory representations of the implied action and predicted the motor consequences by watching a posture without any further kinematic aspect. According to the proposition by van der Kamp and colleagues [35], we assume that visual expertise, based on the ventral system, allowed volleyball athletes to perceive what action the situation affords on the basis of the opponent’s kinematics (e.g., a down-the-line or cross-court shot as the most appropriate action). The involvement of the ventral system is supported by the involvement of temporal areas in observing and reproducing known actions [65,66]. On the contrary, the dorsal system allows athletes to prepare and execute an adequate response action, but it might not be activated in basketball athletes as the shown stimuli did not represent salient sport-related motor primitives for them. A further interpretation might be that extensive visual and motor experience develops a better resonance system specific for trained actions, allowing players to enhance both predictive and anticipatory abilities on the basis of a shared representation between seen actions and similar actions in the sensorimotor repertoire [10]. Indeed, it is known that both visual and motor areas (occipito-temporal and fronto-parietal, respectively) are involved in observation, simulation, and reproduction of a familiar action [62,65,67,68]. Again, basketball players might not interpret the shown action stimuli as they do not represent motor primitives in their discipline. Importantly, both groups of athletes responded equally fast. Therefore, the difference observed in Experiment 1 between volleyball athletes and controls was not responsible for the different cognitive elaboration of the stimuli.

## 4. Conclusions

We used a new approach to study the effect of extensive and intensive visual and motor practice in developing the abilities necessary to predict others’ actions from bodily cues and elaborate motor primitives in sport. In detail, we studied what type of information elite volleyball athletes implicitly extrapolate at hand presentation. Volleyball athletes were chosen because their sport requires highly skilled analyses of the postural information transmitted by the opponents’ hands in order to recognise, in the shortest time possible, the direction of potential attacks and implement the best behavioural responses.

In Experiment 1, to study the effect of the direction of potential attack actions, we presented both oriented hands, simulating a cross-court spike, and frontal hands, simulating a down-the-line spike. The implied action directionality was automatically and covertly extracted only for palm-view hands in volleyball athletes. Interestingly, the ability to read the bodily posture and anticipate the related behavioural response seems to be due to specific expertise and not to sport proficiency in general as basketball athletes, in which spike hand actions are not relevant, did not show any effect (Experiment 2), as did nonathletes controls (Experiment 1).

The sidedness paradigm here used [42] assumes that an attentional shift toward space’s location where the response action will be performed generates a motor preactivation towards that side (see *attention shift hypothesis* [53,54]). Results suggest that volleyball athletes processed a specific action-related index (i.e., the implied spike action directionality) on the basis of their previous visual and motor experiences. Expert athletes may be skilled to code palm-view hands as potentially performing a directional spike action and extract the motor primitives for their sport disciplines to mentally simulate opponents’ actions to anticipate the right receipt action. Indeed, spike actions in volleyball are movements with highly stereotyped spatial and temporal characteristics [69], according to information such as arm trajectory, joint angles, and velocity in extrinsic coordinates (see [70]). Our results suggest that visuomotor expertise might modulate the ability to process some motor primitives (i.e., bodily cues on movement directionality) and develop predictive models concerning the motion dynamics of objects (i.e., the ball) on the basis of hand posture. Elite athletes visually read the sport-relevant kinematics of others’ bodies to anticipate their actions and properly plan an adequate counter-response well before the complete realisation of the observed action. Such predictive abilities are automatic and allow expert athletes to implicitly and unconsciously prepare the congruent behavioural response to anticipate other athletes’ behaviour.

Future directions might see this paradigm applied to volleyball players of different levels of expertise (e.g., athletes playing in different series or with significant years of practice) to investigate whether they differ in their ability to interpret and predict the attack actions and in the effect size. Moreover, such a paradigm might be implemented in both training and player selection in future studies. For example, players might be trained, through sport-specific perceptual–vision training [71,72,73], to read these motor kinematics primitives to improve their decision-making processes and optimise their ability to perceive and process relevant body features [74,75]. Moreover, the paradigm might be expanded to test an athlete’s ability to distinguish between fake and real attack actions according to the level of expertise. Lastly, due to the large effect size observed in this study, the paradigm might be helpful in player selection. The most skilful players are expected to read action directionality according to simple body elements (such as the one used in this study) and show a large effect; however, in general, players with different competitive skills might show a relationship between skillfulness and effect size (more skilful players generate a greater effect size).

## Data Availability

The data presented in this study are available on request from the corresponding author.
